# Do inhibitory receptors need to be proximal to stimulatory receptors to function?

**DOI:** 10.1038/s41435-023-00251-6

**Published:** 2024-01-12

**Authors:** Jonathan D. Worboys, Daniel M. Davis

**Affiliations:** 1 Lydia Becker Institute of Immunology and Inflammation, Faculty of Biology, Medicine and Health, Manchester Academic Health Science Centre, University of Manchester, Manchester, UK; 2 Department of Life Sciences, Sir Alexander Fleming Building, Imperial College London, South Kensington, London, UK

**Keywords:** Signal transduction, Imaging the immune system

We recently demonstrated that the inhibitory receptor T cell immunoreceptor with Ig and ITIM domains (TIGIT) assembles in nanoscale clusters at the T cell surface upon ligation with its ligand CD155. Crucially, these TIGIT-rich nanoclusters co-localise with T cell receptor (TCR) nanoclusters [1], concurrent with reduced effector functions, such as production of the cytokine IL-2 upon superantigen stimulation. TIGIT with mutations that prevented transduction of inhibitory signals via its ITT-like and ITIM domains, still clustered upon ligation with CD155 and localised to TCR clusters, but could not inhibit functional outcomes. Thus, inhibitory TIGIT signalling localised to the TCR leads to less cellular activation. The question arises: is the nanoscale proximity of inhibitory and stimulatory receptors, like TIGIT and the TCR, essential for inhibitory function?

There are two main ways in which proximity of an inhibitory receptor to a stimulatory receptor could be important for functional inhibition: (i) Inhibitory receptors act to disrupt local stimulatory receptor signalling, and/or (ii) Inhibitory receptors require signals from stimulatory receptors to be stimulated themselves. Here, we provide examples with the inhibitory receptors PD-1, CTLA-4 and LAG3 that support each of these views (summarised in Fig. 1).

**Fig. 1:**
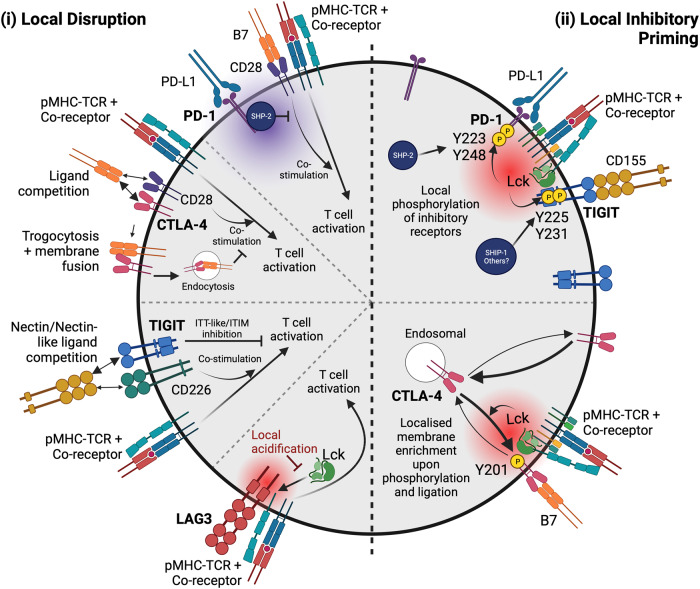
Mechanisms and functions of proximal inhibitory receptors. Inhibitory receptors (in bold) can act locally to disrupt stimulatory receptors preventing cell activation (left) and/or be stimulated to function by local stimulatory signals (right). (i) Local disruption can involve the recruitment of inhibitory molecules (such as the phosphatases SHP-2 binding to PD-1, or SHIP-1 binding to TIGIT) to TCR clusters leading to localised dephosphorylation and inhibition. Local competition for ligand binding may also occur with both CTLA-4 and TIGIT, which share common ligands with stimulatory receptors. CTLA-4 can subsequently internalise and/or degrade its ligands, which further diminish stimulatory signalling of CD28. LAG3 localises to the TCR and its presence can create local acidity that prevents Lck interacting with the co-receptors CD4 and CD8, weakening co-stimulatory signalling. (ii) Inhibitory receptors themselves can be ‘primed’ in a localised manner due to signals they receive at sites of stimulation. Intracellular inhibitory motifs in PD-1 are phosphorylated by Lck, which concentrates at stimulated TCR clusters, leading to the initiation of localised inhibitory signalling. Based on our observations of TIGIT localisation, a similar mechanism likely exists to regulate its inhibitory signalling. Alternatively, inhibitory receptors can be locally recruited from subcellular compartments, such as with CTLA-4. Hubs of Lck activity can favour the selective retention of CTLA-4 at sites of stimulation at the cell membrane, which can promote localised regulatory CTLA-4-B7 interactions. Image created with BioRender.com.

## Evidence that inhibitory receptors are recruited to stimulatory receptors

PD-1 clusters at the immune synapse upon ligation with PD-L1 and PD-L2 [2, 3]. Initially, it was observed that PD-1 clusters co-localised with TCR clusters in murine T cells early in immune synapse formation [2]. Later, this model was refined as it was demonstrated that PD-1 better co-localises with the co-stimulatory molecule CD28 throughout the maturation of the immune synapse [3]. CTLA-4 constantly traffics from subcellular vesicles to the membrane but is predominantly localised in subcellular compartments. Both TCR stimulation and interaction with B7 ligands in trans, causes CTLA-4 accumulation and clustering at the immune synapse [4, 5]. CTLA-4 clustered proximally to TCR early in synapse formation, and associated with the peripheral synaptic region when the TCR concentrates to a central cluster at later timepoints. Likewise, LAG3 also clusters with the TCR at the immune synapse, in a ligand-independent manner [6]. Thus, several different types of inhibitory receptors are specifically recruited to activating receptors at immune synapses.

## Evidence that inhibitory receptors act locally to disrupt stimulatory signalling

PD-1 ligation with PD-L1 or PD-L2 leads to phosphorylation of PD-1 [2], which can then recruit the phosphatase SHP-2, leading to dephosphorylation of proximal CD28 molecules and inactivation of CD28 signalling [3]. CTLA-4 at the synapse can also reduce proximal CD28 signalling [5], likely mediated by both competing for B7 ligand binding in trans and internalisation of trogocytosed B7 ligands through either *trans* or *cis*-endocytosis, which could be enhanced by co-proximity [7, 8]. Proximal disruption by inhibitory receptors does not necessarily require phosphorylation-mediated signalling. Accumulation of LAG3 at TCR complexes increased its proximity to the co-receptors CD4 and CD8, which led to localised acidification through its glutamic acid-proline dipeptide repeat (EP motif), in turn disrupting Lck-CD4 or Lck-CD8 interactions and subsequent co-stimulation [6].

## Evidence that inhibitory receptors require local stimulation to function

Chimeric versions of PD-1 that contained different numbers of Ig domains in its extracellular tail had differing inhibitory potential, conistent with the kinetic-segretaion model of positioning proteins at the immune syanpse according to their size [2]. PD-1 with large extracellular domains were excluded from TCR clusters and could not prevent downstream TCR signalling and IL-2 secretion. Additionally, PD-1 phosphorylation only occurred when PD-1 was ligated and colocalised with the TCR, which correlated with SHP-2 recruitment. This is evidence that PD-1 proximity to the TCR is critical to initiate functional inhibitory signalling. This is not limited to T cells, as inhibition by Killer Ig-like receptors required proximity to the activating receptor NKG2D at the surface of human NK cells, which could also be perturbed by altered protein size [9].

Other evidence is that TCR stimulation leads to an accumulation of CTLA-4 at the immune synapse in a manner dependent on the TCR signalling strength [4]. The Src-family kinase, Lck, can phosphorylate cytoplasmic CTLA-4 tyrosine residues which promotes its localisation from subcellular vesicles to the membrane [10]. Stimulation of the TCR generates hubs of Lck activity at the immune synapse that could lead to localised surface enrichment of CTLA-4 where it can bind to B7 ligands to provide negative feedback.

## Concluding remarks

Providing inhibitory signals to cells that do not require inhibition would feasibly be wasteful of cellular activity and resources. This could provide an evolutionary rationale for inhibitory signalling to only act where and when it is necessary. Often, textbook diagrams depict receptor transduction occurring solely upon ligation, but this is too simplistic as inhibitory receptor signalling is context specific. Initiating inhibitory processes likely requires signals from local stimulatory receptor signalling hubs, as is the case for PD-1, CTLA-4 and potentially for TIGIT. In some cases, inhibitory receptors may not require signalling to function as their proximity to stimulatory receptors by itself can be inhibitory, as with LAG3. Limiting the ability of inhibitory receptors to function at precise nanoscale locations of stimulation permits a spatiotemporal regulation governed by stimulatory signals, providing highly efficient regulatory mechanisms.

## References

[1] Worboys JD, Vowell KN, Hare RK, Ambrose AR, Bertuzzi M, Conner MA, et al. TIGIT can inhibit T cell activation via ligation-induced nanoclusters, independent of CD226 co-stimulation. Nat Commun. 2023;14:5016. 10.1038/s41467-023-40755-3. 37596248 10.1038/s41467-023-40755-3 PMC10439114

[2] Yokosuka T, Takamatsu M, Kobayashi-Imanishi W, Hashimoto-Tane A, Azuma M, Saito T. Programmed cell death 1 forms negative costimulatory microclusters that directly inhibit T cell receptor signaling by recruiting phosphatase SHP2. J Exp Med. 2012;209:1201–17. 10.1084/jem.20112741. 22641383 10.1084/jem.20112741 PMC3371732

[3] Hui E, Cheung J, Zhu J, Su X, Taylor MJ, Wallweber HA, et al. T cell costimulatory receptor CD28 is a primary target for PD-1-mediated inhibition. Science. 2017;355:1428–33. 10.1126/science.aaf1292. 28280247 10.1126/science.aaf1292 PMC6286077

[4] Egen JG, Allison JP. Cytotoxic T. lymphocyte antigen-4 accumulation in the immunological synapse is regulated by TCR signal strength. Immunity. 2002;16:23–35. 10.1016/s1074-7613(01)00259-x. 11825563 10.1016/s1074-7613(01)00259-x

[5] Yokosuka T, Kobayashi W, Takamatsu M, Sakata-Sogawa K, Zeng H, Hashimoto-Tane A, et al. Spatiotemporal basis of CTLA-4 costimulatory molecule-mediated negative regulation of T cell activation. Immunity. 2010;33:326–39. 10.1016/j.immuni.2010.09.006. 20870175 10.1016/j.immuni.2010.09.006

[6] Guy C, Mitrea DM, Chou PC, Temirov J, Vignali KM, Liu X, et al. LAG3 associates with TCR-CD3 complexes and suppresses signaling by driving co-receptor-Lck dissociation. Nat Immunol. 2022;23:757–67. 10.1038/s41590-022-01176-4. 35437325 10.1038/s41590-022-01176-4 PMC9106921

[7] Qureshi OS, Zheng Y, Nakamura K, Attridge K, Manzotti C, Schmidt EM, et al. Trans-endocytosis of CD80 and CD86: a molecular basis for the cell-extrinsic function of CTLA-4. Science. 2011;332:600–3. 10.1126/science.1202947. 21474713 10.1126/science.1202947 PMC3198051

[8] Xu X, Dennett P, Zhang J, Sherrard A, Zhao Y, Masubuchi T, et al. CTLA4 depletes T cell endogenous and trogocytosed B7 ligands via cis-endocytosis. J Exp Med. 2023;220:e20221391. 10.1084/jem.20221391. 37042938 10.1084/jem.20221391 PMC10103642

[9] Köhler K, Xiong S, Brzostek J, Mehrabi M, Eissmann P, Harrison A, et al. Matched sizes of activating and inhibitory receptor/ligand pairs are required for optimal signal integration by human natural killer cells. PloS one. 2010;5:e15374. 10.1371/journal.pone.0015374. 21179506 10.1371/journal.pone.0015374 PMC3001952

[10] Bradshaw JD, Lu P, Leytze G, Rodgers J, Schieven GL, Bennett KL, et al. Interaction of the cytoplasmic tail of CTLA-4 (CD152) with a clathrin-associated protein is negatively regulated by tyrosine phosphorylation. Biochemistry. 1997;36:15975–82. 10.1021/bi971762i. 9398332 10.1021/bi971762i

